# Dimerization of lipocalin allergens

**DOI:** 10.1038/srep13841

**Published:** 2015-09-08

**Authors:** Merja H. Niemi, Marja Rytkönen-Nissinen, Ilja Miettinen, Janne Jänis, Tuomas Virtanen, Juha Rouvinen

**Affiliations:** 1 Department of Chemistry and Biocenter Kuopio, University of Eastern Finland, PO BOX 111, 80101 Joensuu, Finland; 2 Department of Clinical Microbiology, Institute of Clinical Medicine and Biocenter Kuopio, University of Eastern Finland, PO BOX 1627, 70211 Kuopio, Finland; 3 Institute of Dentistry, School of Medicine, University of Eastern Finland, PO BOX 1627, 70211 Kuopio, Finland

## Abstract

Lipocalins are one of the most important groups of inhalant animal allergens. The analysis of structural features of these proteins is important to get insights into their allergenicity. We have determined two different dimeric crystal structures for bovine dander lipocalin Bos d 2, which was earlier described as a monomeric allergen. The crystal structure analysis of all other determined lipocalin allergens also revealed oligomeric structures which broadly utilize inherent structural features of the β-sheet in dimer formation. According to the moderate size of monomer-monomer interfaces, most of these dimers would be transient in solution. Native mass spectrometry was employed to characterize quantitatively transient dimerization of two lipocalin allergens, *Bos d 2* and *Bos d 5,* in solution.

A characteristic feature for a type I allergy is the development of a special class of IgE antibodies against a group of proteins which are called allergens^1^. It has been observed that known allergens are members of a limited class of protein families, which suggests that structural features of these proteins would contribute to allergenicity. It is also known that the immune system develops IgE antibodies in parasitic worm (helminths) infections. Recently, structural resemblances between allergens and helminth antigens^2^ have been pointed out. In immediate phase reaction allergens, cross-link allergen-specific IgE antibodies bind to the FcεRI receptors on the surface of mast cells or basophils. This leads to the degranulation and release of pharmacologically active mediators, such as vasoactive amines.

The cross-linking requires that the allergen is at least bivalent, displaying two conformational B-cell epitopes to which IgE antibodies would bind. If the allergen is monomeric and thus asymmetric, this would require two different IgE antibodies and, consequently, two different epitopes for the allergen. However, if the allergen is oligomeric this would allow a multifold presentation of a monovalent epitope and, consequently, requires only one allergen-specific IgE antibody. We have previously characterized some common allergens and suggested that oligomerization is a common feature among allergens. Many allergens are transient dimers in solution, whose monomer-dimer ratio is dependent on protein concentration and the dissociation constant (*K*_D_)^3^. In order to investigate the prevalence of dimerization, we have studied lipocalins, one of the major allergen families.

Lipocalins include the most important inhalant animal allergens. In addition, a bovine milk allergen (*Bos d 5*) and several arthropodan allergens are lipocalins. Lipocalins are typically small proteins (about 200 amino acid residues) composed of an 8-stranded up-and-down antiparallel β-barrel and one α-helix. There is a hydrophobic cavity inside the β-barrel capable of binding a hydrophobic or amphiphilic ligand (Fig. 1a). In most cases, the native ligands for lipocalins are unknown^4^,^5^. In order to analyze the oligomeric structures of lipocalins, we have studied all 10 crystal structures of lipocalin allergens in the Protein Data Bank (PDB). Bovine dander allergen *Bos d 2* was the only one found to be monomeric^6^. Because the protein had been crystallised at an acidic pH (3.4–4.5)^7^, we decided to search for a new crystal form at a more neutral pH. In fact, both new crystal forms of *Bos d 2* contained a symmetric weak dimer. The existence of a dimeric *Bos d 2* in solution was observed by native mass spectrometry (MS). Furthermore, we used native MS to quantify the dimerization for *Bos d 2* and *Bos d 5*. In the following, we present an analysis of oligomeric structures of lipocalin allergens and discuss their implications for the presentation of multiple epitopes and the triggering of FcεRI bound IgE antibodies on mast-cells and basophils.

## Results

### Dimeric crystal structures of Bos d 2

**Figure 1:**
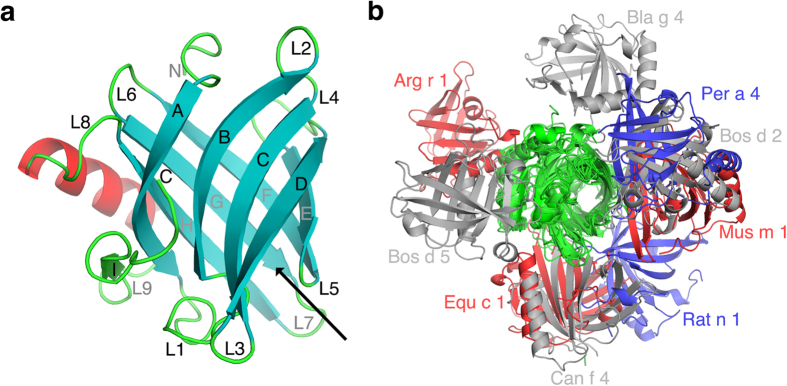
(**a**) The ribbon representation for monomeric *Bos d 2.* The eight antiparallel β-strands (**A**–**H**) forming a central barrel are shown as cyan arrows. The β-strand (I) outside the barrel is in green. The α-helix is in red and loops connecting different secondary structure elements are labelled L1 to L8. The black arrow shows the entrance to the ligand binding pocket (**b**) The monomers of 9 different lipocalin allergens which form symmetric homodimers are superimposed to observe the orientation of the second monomer. The superimposed monomers are in green and grey, blue and red colours are used for the second monomers.

**Figure 2:**
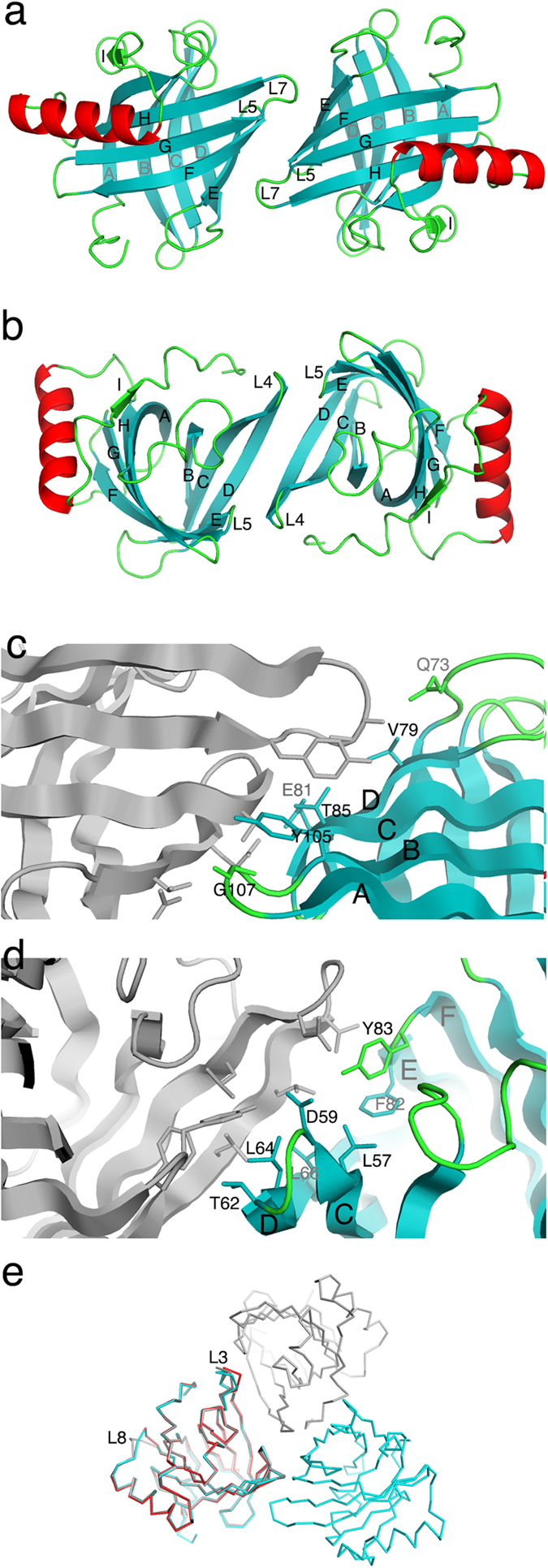
Dimerization of *Bos d 2* observed in crystals as a ribbon presentation. (**a**) The symmetric dimer of *Bos d 2* observed in the monoclinic (C2) crystal form, (b) in the trigonal (P3_2_21) crystal form. The key residues which contribute to the binding on the monomer-monomer interface in *Bos d 2* dimer in monoclinic (**c**) and in trigonal (**d**) crystal forms. (**e**) The superimposition of a Cα-backbone of monomeric *Bos d 2* (orthorhombic, in red), dimeric *Bos d 2* (monoclinic, in cyan), and dimeric *Bos d 2* (trigonal, in grey).

The search for new crystal forms for bovine allergen *Bos d 2* at neutral pH resulted in two new crystal forms. Monoclinic crystals diffracted at a 1.4 Å resolution. The asymmetric unit contained one *Bos d 2* molecule but the space group C2 includes a 2-fold symmetry axis, consequently, half of the dimer can be formed by a crystallographic symmetry operator. Trigonal crystals diffracted at 1.75 Å resolution. Again, the asymmetric unit contained one *Bos d 2* molecule and a dimer can be formed with the use of a crystallographic symmetry operator. *Bos d 2* contains a central β-barrel composing of eight antiparallel β-strands (A-H). In addition, there is a short β-strand (I) as well as an α-helix (Fig. 1a). The r.m.s. differences of *Bos d 2* molecules in orthorhombic and monoclinic crystals is 0.226 Å, between orthorhombic and triclinic crystals there is 0.297 Å and between monoclinic and triclinic crystals there is 0.242 Å, which are in the range observed for different molecules in the asymmetric unit in protein crystal structures. The largest changes in the structures are located in the L3 loop and the L8 loop that connects the C-terminus of β-strand H to the α-helix (Fig. 2e).

The monomer-monomer interface area for the dimer of *Bos d 2* in a monoclinic crystal is 389 Å^2^. The interface consists of a terminus of β-strands E-H and loops L5 and L7 (Fig. 2a). Amino acid residues Thr85, and Tyr105 along the 2-fold axis pack against the same residues from the second monomer (Fig. 2c). Glu81, located on the interface, has multiple conformations. In addition, Gln73 makes a hydrogen bond with the main-chain carbonyl of Gly107, and the side-chain of Val79 packs against Gly107. The interface area between monomers in the trigonal crystal is also 389 Å^2^ but there are many more hydrophobic contacts on the monomer-monomer interface. The side-chains of Thr62, Leu64 and Leu66 from the β-strand D form the core of the interface. In addition, the residues Phe82 and Tyr83 from the L5 loop, as well as Leu57 from the β-strand C, are located on the interface (Fig. 2b,d). Because there is a clearly higher number of hydrophobic residues on this interface, we assume that this interface is more favourable in solution than the dimer observed in monoclinic crystal form.

### Analysis of crystal structures of lipocalin allergens

In addition to *Bos d 2*, there are three-dimensional coordinates for nine other allergens in the PDB. Seven allergens are from mammals and three from arthropods (Table 1).

**Figure 3:**
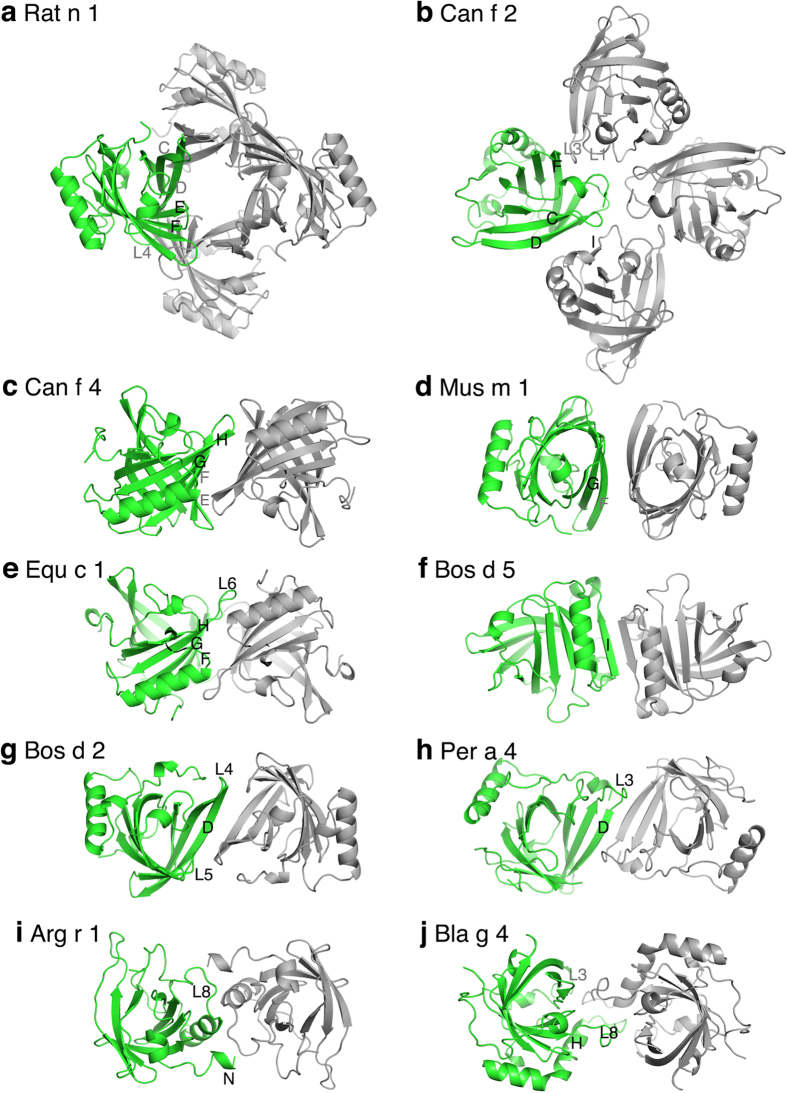
The oligomeric structures for 10 lipocalin allergens observed in crystals. One monomer is in green, other monomers in grey. The structural elements forming monomer-monomer interfaces are labelled.

*Rat n 1* allergen is a major urinary protein of male rats. The tetrameric structure of Rat n 1 has been reported in monoclinic and orthorhombic crystal forms^8^,^9^. The structure contains a complete tetramer with 222 symmetry, each monomer packs against two other monomers having two different interfaces. The larger monomer-monomer interface (708 Å^2^) is formed by strand D-F and especially by the L4 loop between β-strands D and E (Fig. 3a). Three aromatic hydrophobic residues Tyr72, Phe81, and Phe108 can be found on the interface, which suggests strong hydrophobic interactions, which would favour oligomer formation. The second interface is smaller (457 Å^2^) and formed mainly by a β-strand C. Tyr86 from this β-strand packs with the same residue from the second monomer. Because the second interface is smaller with fewer hydrophobic interactions, this would indicate that tetramer dissociates easily to dimers at lower protein concentrations.

*Can f 2* allergen has been reported to be a dimeric protein^10^ but, when describing the crystal structure, Madhurantakam *et al.* consider that *Can f 2* is monomeric in neutral pH^11^. A symmetric dimer has been described with a 447 Å^2^ interface area^12^. However, the same crystal structure also shows a tetrameric arrangement in which one interface (576 Å^2^) is formed between β-strands C and D from one monomer and β-strand I and the α-helix from the second monomer (Fig. 3b). Phe72 on the outer surface of the β-strand D packs with the hydrophobic Leu131 and Val50 from the second monomer. The second interface is smaller (380 Å^2^) and it is formed between N-terminal residues and the C-terminus of β-strand F from one monomer and loops L1 and L3 from the second monomer. Trp35 from loop L1 interacts with Val95 and Pro97 from another monomer. There is no symmetry axis between these interfaces but there is one 2-fold axis through the whole tetramer (Fig. 3b).

*Can f 4* is the second dog lipocalin allergen, whose crystal structure has been determined at 2.6 Å resolution. The asymmetric unit contained one monomer and one symmetric dimer in which the monomer-monomer interface area is 640 Å^2^. Hydrophobic residues located on β-strands E-H form the core of the dimeric interface (Fig. 3c). These include Leu76, Tyr86, Val102, Val104, and Ile110^12^.

*Mus m 1* is a mouse major urinary protein, whose structure was determined at 2.4 Å resolution and on the basis of the crystal structure it was deduced to be a dimer^8^. The monomer-monomer interface area is smaller than other lipocalins allergens (389 Å^2^) but it contains a cluster of hydrophobic residues, Leu 52, Val57, and Val74, as well as a salt-bridge (Lys59-Asp76), which are all located in β-strands F and G (Fig. 3d).

The crystal structure for *Equ c 1* was determined at 2.3 Å resolution with one molecule in the asymmetric unit^13^. The monomer-monomer interface area is the largest among the lipocalin allergens (1025 Å^2^). As in *Can f 4,* the interface is formed by β-strands F, G, and H but loop L6 has a larger role on the interface (Fig. 3e). The hydrophobic residues Val93, Phe112, Y108, and V110 are located on this interface.

*Bos d 5,* or β-lactoglobulin, is a major protein in bovine milk (concentration ca. 3–4 mg/ml). On the basis of its crystal structure, *Bos d 5* has regularly been described as a dimer, although the interface area is only 528 Å^2^. The structure has been solved at 1.8 Å resolution with one dimer in the asymmetric unit^14^. Dimerization is achieved by main-chain hydrogen bonds between β-strands I and participation of some N-terminal residues (Fig. 3f). Hydrophobic residues Ile29, Ile147, Leu139 and Phe151 can be found on the interface. *Bos d 5* also existed as a dimer in complex with Fab fragments of an IgE antibody^15^.

*Per a 4* is a lipocalin allergen from the American cockroach (*Periplaneta americana*). On the basis of gel-filtration, it was deduced that *Per a 4* would exist as a dimer in solution. The proposed dimer consisted of both molecules in the asymmetric unit (interface 663 Å^2^), having some hydrophobic contacts, but the dimer is asymmetric^16^. However, the crystal structure also shows a two-fold symmetry axis between the two molecules in the asymmetric unit. The interface area for this dimer is smaller (403 Å^2^), but there is a cluster of hydrophobic Ile65 and Ile69 from the β-strand D and loop L3. In addition, there are main-chain hydrogen bonds between β-strands D (Fig. 3h).

*Arg r 1* is an allergen from the European pigeon tick, whose bite can cause anaphylactic reactions in sensitized humans^17^. The crystal structure of *Arg r 1* has been determined at high resolution (1.4 Å) but there is no structure paper. The crystal structure contains three molecules in the asymmetric unit in which two molecules form a dimer and the third one forms a similar dimer with a crystallographically symmetric molecule. The monomer-monomer interface is 530 Å^2^. The residues from the N-terminal and the L8-loop between the β-strand H and the α-helix are located on the interface (Fig. 3i). There are four hydrogen bonds between the main-chains and other polar contacts but not very many hydrophobic contacts. However, the existence of two similar dimers in crystal suggests that this dimer is quite favourable.

*Bla g 4* is also a cockroach allergen but from a different species (*Blattella germanica*). *Bla g 4* is assumed to be a monomer in solution, according to gel-filtration results^16^. However, two molecules in the asymmetric unit form a dimeric structure having a quite large monomer-monomer interface (842 Å^2^), which is formed by a L8-loop between the β-strand H and the α-helix and L3-loop (Fig. 3j). A large number of hydrophobic residues, Ile46, Met 127, Leu 129, Phe 132, Met141, and Ile142, on the interface support the dimer formation.

### Comparison of lipocalin allergen oligomeric structures

All the oligomeric structures of lipocalin allergens are different to each other. This is probably due to the fact that amino acid identity between them is generally low (5–20%). The highest identity is between *Mus m 1* and *Rat n 1* allergens (64%) but they still have different oligomeric structures. Different oligomerization can be explained by variation of amino acid residues on the protein surfaces. Dayhoff *et al.* have observed that oligomers would have the same binding mode if sequence identity would be 70% or higher between them^18^. In order to see different modes for dimerization, we have superimposed the symmetric dimeric structures of allergens in a way that the position for one monomer is fixed (Fig. 1b). Although the position of the second monomer varies considerably, there are no monomers bound at both ends of the central β-barrel. Consequently, the oligomerization would not prevent ligand binding or release. Dimerization of *Bos d 2* and *Per a 4* resemble each other: both lipocalins form an extended β-sheet through β-strand D, although there are also some differences in the packing of these β-strands. In addition, the dimers of *Can f 4* and *Equ c 1* are quite similar: they both have a similar packing of β-sheets together^15^. We can also notice that the majority of the lipocalin allergens utilize inherent features of a β-sheet in the formation of symmetric dimers. *Bos d 5*, *Bos d 2*, and *Per a 4* form an extended β-sheet by joining the β-sheets from two monomers together. In *Bos d 2* and *Per a 4,* this is mediated by β-strand D, in *Bos d 5* by β-strand I. On the other hand, in dimerization, *Can f 2, Can f 4, Equ c 1, Mus m 1,* and *Rat a 1* form a new β-sandwich structure by bringing β-sheets from two monomers together. In addition, the monoclinic structure of *Bos d 2* utilizes similar β-sheet packing. The exceptions are oligomeric structures of *Bla g 4*, and *Arg r 1*, which are predominantly joined together by long L8 loops. The observation that β-sheets have an essential role in the formation of dimers suggests that the inherent features of β-sheets (packing of β-strands and extension of β-sheets) also have a role in the formation of weak dimers and that there is a limited number of arrangements to form transient dimers. In consequence, the packing features at β-sheets can perhaps also be used to analyze different protein-protein interfaces in crystals in order to distinguish native interactions and crystal contacts.

### Native mass spectrometry of lipocalin allergens

The accurate molecular mass of recombinant *Bos d 2* (*rBos d 2*) was determined by high-resolution Fourier transform ion cyclotron resonance (FT-ICR) mass spectrometry in denaturing solution conditions (data not presented). The experimentally determined molecular mass was 17845.89 ± 0.03 Da, which agrees perfectly with the theoretical mass calculated from the amino acid sequence of a mature *Bos d 2* with two disulfide bonds (17845.81 Da). Apart from the main proteoform, the mass spectrum revealed that a substantial amount of the protein molecules contained a pyrrolidone carboxylic acid (PCA), formed through a cyclization of the N-terminal glutamine residue (17828.90 Da). Repeated measurements showed that the PCA-containing proteoform started to dominate during long-term storage. The sequence of *rBos d 2* contains two methionine residues. The analysis also revealed that the protein was partly oxidized, probably through a single methionine residue and thus displayed a +16 Da mass difference when compared to the main proteoform (17861.90 Da).

**Figure 4:**
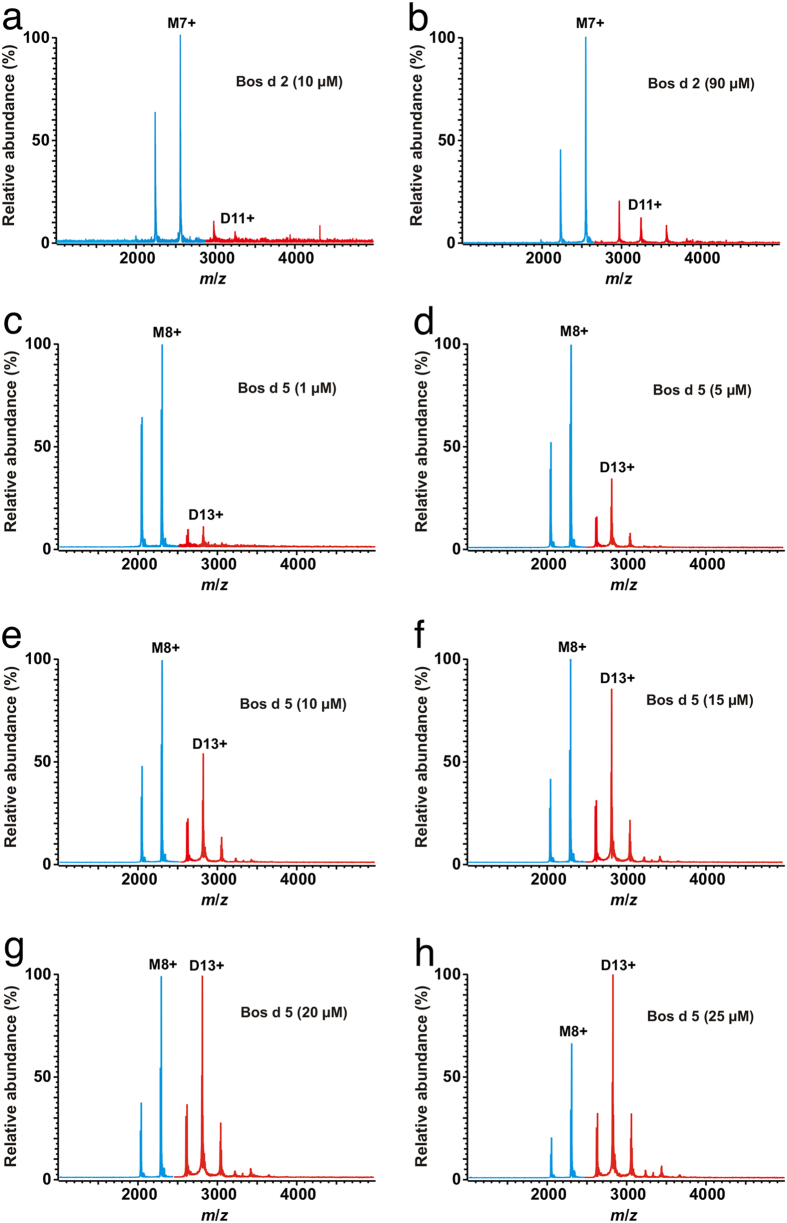
Native mass spectra of lipocalin allergens at different protein concentrations. (**a**,**b**) *rBos d 2* at protein (monomer) concentrations of 10 and 90 μM. The peaks representing the protein monomer (M) are in blue, while the peaks representing the protein dimer (D) are in red. The most abundant peaks for the monomer and the dimer have been assigned with the number indicating the ion charge state (i.e., M7+ = [*rBos d 2* + 7H]^7+^). **(c-h)** Native mass spectra of *Bos d 5* at a protein (monomer) concentration of 1-25 μM.

**Figure 5:**
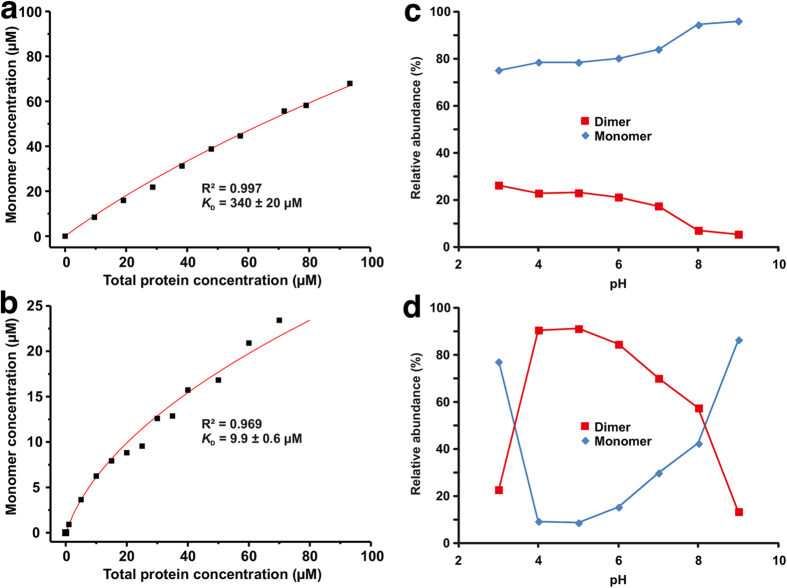
Protein monomer concentration as a function of total protein concentration for (a) *rBos d 2* and (b) *Bos d 5* as calculated from the native mass spectra. The solid lines are the best fits of the data to Equation 4 (see, Online methods for details) for determining *K*_D_ value for dimerization. Relative abundances of the protein monomer and the dimer for (**c**) *rBos d 2* and (**d**) *Bos d 5* depending on solution pH at a protein (monomer) concentration of 40 μM.

Native MS was used to study the dimer formation of *rBos d 2* in native-like solution conditions (i.e., 10 mM ammonium acetate, pH 6.9). The basic protocol for native MS was similar to our previous work^3^, which allows for the weak transient protein dimers to be detected. The native MS spectrum of *rBos d 2* (10 μM in respect to the protein monomer) at pH 7 showed signals of both monomers and dimers in solution (Fig. 4a). When the protein concentration was increased to 90 μM, the relative intensity of the dimer increased accordingly (Fig. 4b). These results indicate that *rBos d 2* is indeed a weak transient dimer in solution. To further quantify the dimerization (see Methods for details), we obtained protein monomer–dimer ratios from the native mass spectra over a wide range of protein (monomer) concentrations (0–90 μM). The fitted curve of the free monomer concentration against the total protein concentration is presented in Fig. 5a. The determined value for the equilibrium dissociation constant (*K*_D_) for self-association was *K*_D_ = 340 ± 20 μM. We also analyzed the pH-dependence of the dimerization (Fig. 5c), and the amount of *rBos d 2* dimer never exceeded the amount of the monomer. The amount of dimer slowly decreased when the pH changed to be more alkaline. The maximal amount of dimer was present at around pH 3.

For comparison and validation, we analyzed bovine milk β-lactoglobulin (*Bos d 5*), another dimeric lipocalin allergen, by native MS in a range of protein monomer concentrations (Fig. 4c–h). The determined equilibrium constant for *Bos d 5* self-association was *K*_D_ = 9.9 ± 0.6 μM (Fig. 5b). Thus, *Bos d 2* is clearly a weaker transient dimer than *Bos d 5*. It must be noted that the *K*_D_ determined for *Bos d 5* represents an apparent value, as there is a mixture of three dimers in solution (AA, AB and BB), due to the presence of two natural variants, A and B. We did not attempt to measure individual *K*_D_s for AA, AB and BB, since this would require solving a complex set of equilibrium equations, and the native MS data may not be sufficient to derive accurate values for all three *K*_D_ values. Moreover, the apparent *K*_D_ reflects better the situation for natural *Bos d 5*, which is in dynamic equilibrium between A, B, AA, AB, and BB. However, relative intensities suggest that AA forms a somewhat weaker transient dimer (i.e., higher *K*_D_) than BB. Furthermore, the pH profiles for the dimerization between *rBos d 2* and *Bos d 5* were slightly different. The amount of *Bos d 5* dimer was highest at pH 4–5 (>90%) and it continuously decreased from pH 5 to 8 (Fig. 5d). Above pH 8, the amount of dimer decreased rapidly, due to protein denaturation. Also, the amount of dimer rapidly decreased at pH < 4. The results are in line with the results of Invernizzi *et al.*^19^.

Because of its easy availability, biophysical properties of *Bos d 5* have been studied extensively. Dimerization of *Bos d 5* has been studied by several different analytical methods, including isothermal titration calorimetry (ITC), analytical ultracentrifugation (UAC) and native MS^19^,^20^,^21^,^22^,^23^,^24^. It has been shown that *Bos d 5* is a transient dimer in a wide range of solution conditions. For example, the *K*_d_ values for the two variants A and B, determined by dilution-ITC at 20 °C and pH 7, are 24 and 14 μM, respectively. This is fully consistent with our present data, as well as our earlier work^3^. However, dimerization of *Bos d 5* is highly dependent on pH, ionic strength and temperature, and a range of different *K*_d_ values (∼1–200 μM) have been reported for both the variants in different conditions. Most studies have dealt with either of the natural variants of *Bos d 5*, A or B. However, natural *Bos d 5* exists as a mixture of these two variants, and the predominant dimer in solution is a heterodimer, AB^3^. The apparent *K*_D_ of 9.9 μM for *Bos d 5*, determined in this work is slightly lower than the values determined earlier for both variants by using dilution-ITC^20^,^21^, but clearly higher than determined for the A variant (*K*_D_ = 2.2 ± 0.7 μM) by Liu *et al.* using native MS^23^. There is also evidence that higher oligomeric states (e.g., octamers) can form around a pH close to the isoelectric point of the protein (∼4.6) and at low temperature^25^. Based on the dilution-ITC data, the dimerization of *Bos d 5* is both enthalpy- and entropy-driven, although the enthalpy contribution is more dominant for the association free energy in the variant A^22^. This is consistent with the monomer-monomer interface formed through both hydrogen bonds, as well as with hydrophobic interactions.

We have earlier introduced the use of native mass spectrometry into studying dimerization of proteins in solution^3^,^15^ and develop it further in this study. The obtained results for *Bos d 5* are in good agreement with the literature. The results suggest that native MS is able to detect and even quantify transient dimerization of proteins in solutions having *K*_D_ values in the high-μM to low-mM range.

## Discussion

Proteins which exist in a monomer-dimer equilibrium in solution can be defined as weak or transient dimers. Both the *K*_d_ value for the dimer and the protein concentration affect the degree of oligomerization (i.e., whether the protein is preferentially in the dimeric or the monomeric form). In addition, ionic strength and pH can play significant roles as well, and temperature markedly affects those interactions that are mainly entropy-driven. Typically, *K*_d_s are in the micromolar range for transient dimers when the monomer-monomer interface is less than 1000 Å^2^ 26. Transient protein oligomerization is difficult to study experimentally. For example, size-exclusion chromatography (SEC) may suffer from protein interactions with the column matrix, which results in an erroneous estimation of molecular weight and can also shift the equilibrium towards a monomeric form^27^. Dynamic/static light scattering (DLS/SLS) or low angle X-ray scattering (SAXS) have not been widely used in the study of transient dimers, probably due to the challenges in measuring mixtures of different sizes of particles in solution. ITC and AUC are better suited for the purpose, and ITC also directly provides estimates of the association enthalpy and entropy. However, ITC suffers from rather low sensitivity and requires high intial concentrations (for dilution-ITC experiments). Furthermore, AUC requires *a priori* knowledge of the oligomeric state for the model used to fit the data. Native MS has several benefits over the other techniques; it provides direct stoichiometric data on different oligomeric forms present simultaneously in solution, is highly sensitive, and also provides quantitative data for the estimation of *K*_d_ values. It is also not inherently affected by association/dissociation kinetics, like many other techniques, since it measures a time-averaged thermodynamic equilibrium. Recently, native MS has been utilized in the determination of *K*_d_ values for *Bos d 2* and *Bos d 5* (340 and 9.9 μM; this study) and *Can f 4* (27 μM)^15^ allergen dimers. All these values of *K*_D_ are in the micromolar range, typical for transient dimers. The size of the monomer-monomer interfaces varies between 389 Å^2^ to 1025 Å^2^ in the 10 studied lipocalin allergen crystal structures (Table 1) suggesting all these dimers could be transient^26^. It also seems that *Equ c 1*, which has the largest monomer-monomer interface, is a transient dimer because it was suggested that *Equ c 1* exists as a mixture of monomers and dimers in solution^28^.

The capability of allergens to form dimers or higher oligomers could have a remarkable effect on mast-cell triggering, because a monovalent allergen could present a single epitope two or more times and only a single allergen specific IgE antibody is needed. In theory, if an allergen forms a dimer, only one monoclonal IgE antibody is needed for mast-cell triggering. In consequence, allergen oligomerization could decrease the need for polyclonal IgE antibodies, which, in turn, would have implications on the nature of the epitope repertoire of the IgE response^29^.

**Figure 6:**
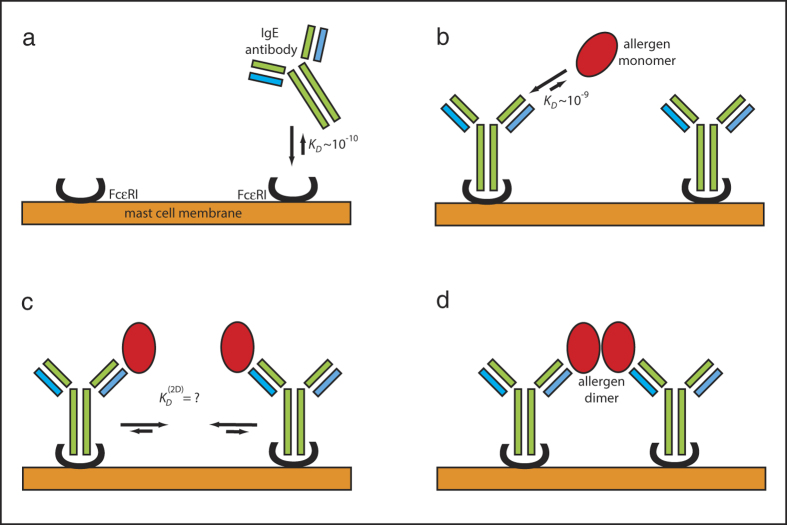
The sketch for the initial events leading to the allergen triggered signal transduction. (**a**) allergen-specific IgE serum antibodies bind to FcεRI receptors on the surface of mast cells or basophils with high affinity. (**b**) allergen exposure leads to a binding of monomeric allergens to specific IgE antibodies already bound to FcεRI receptors. (**c**) tethering of monomeric allergens on the cell surface results in dimerization of allergen monomers. The 2-dimensional dissociation constant for allergen dimers on the cell surface is not known. (**d**) the allergen dimerization has cross-linked FcεRI bound IgE antibodies that lead to the signal transduction.

If we sketch the initial process leading to the mast-cell degranulation we can first note that serum IgE antibodies bind very strongly to the FcεRI receptors on the surface of mast cells or basophils (*K*_D_ ∼ 0.1 nM) (Fig. 6a). In addition, the binding of allergens to IgE antibodies is also potent (*K*_D_ ∼ 0.1–1.0 nM) (Fig. 6b), generally more potent than antigen binding to IgG antibodies^1^,^15^. Both these interactions are many order of magnitudes stronger than transient dimerization of allergens (*K*_D_ ∼ 10 μM) in solution. Therefore, we can assume that IgE antibodies are predominantly bound to the FcεRI receptors on the cell surface and the allergen prefers to bind as a monomer to the surface bound specific high-affinity IgE-antibodies (Fig. 6b). We could consider that this process leads to colocalization^30^ of allergens, thus they increase their “concentration” on the surface of mast cells, allowing the dimerization to occur (Fig. 6c,d).

In fact, allergen dimerization on surfaces of mast cells has a clear resemblance to many other signal transduction processes. In a recent study, dimerization of a lipid-anchored H-Ras protein on the membrane surfaces was studied with a combination of biophysical methods and the *K*_D_^(2D)^ for the dimer was found to be about 1000 molecules/μm^2^. The authors estimated that this would correspond to *K*_D_ being ∼500 μM in solution^31^, which indicates a clearly weaker dimer than lipocalin dimers. It is clear that in order to prove the role of allergen dimerization for the clustering of FcεRI bound IgE antibodies on the surface of mast cells or basophils substantial further experimentation on the membrane surfaces is required. This study, which is based on the analysis of three-dimensional structures of allergens and their cabability to form dimers in solution, should be considered as the first step in continuing efforts to understand the molecular basis of allergenicity and how structural features of allergens may be related to the signal transduction processes.

## Methods

### Mass spectrometry of Bos d 2 and Bos d 5

Recombinant *Bos d 2* was expressed and purified as reported earlier^7^. Native *Bos d 5* from bovine milk was obtained as a lyophilized powder from Sigma-Aldrich (St. Louis, MO, USA). All mass spectrometric analyses were carried out by a 12 T APEX-Qe FT-ICR mass spectrometer (Bruker Daltonics, Billerica, MA, USA), equipped with an ESI source (Apollo-II). The analysis in denaturing solution conditions was performed with a *rBos d 2* sample diluted to 2 μM with MeCN:H_2_O:HOAc (49.5:49.5:1.0, v/v) and the solution was injected into the ion source by a syringe pump with a flow rate of 1.5 μl/min. Nitrogen was used as the drying (250 °C, 6 mbar) and nebulizing gas. Ions from an ESI-source were accumulated in the hexapole ion trap for 1.0 s and transferred to the ICR cell for trapping, excitation and detection. For a mass spectrum, a total of 256 co-added (1 Mword) time-domain transients were recorded and subjected to fast Fourier transform and magnitude calculation. Mass spectra were externally calibrated by using the ions of an ES Tuning Mix (Agilent Technologies, Santa Clara, CA, USA). For native MS experiments, protein samples were desalted and exchanged into a 10 mM ammonium acetate buffer (pH 6.9) with the PD-10 columns (GE Healthcare Bio-Sciences, Uppsala, Sweden). The instrumental parameters were carefully optimized to maintain weak non-covalent interactions in the gas-phase and to avoid any unintentional oligomer dissociation upon ionization/desolvation^3^. A data set contained 300 co-added, 256 kword time-domain transients. The dimerization of *Bos d 2* and *Bos d 5* were studied in the pH range 3–9. Proteins were measured at a (protein monomer) concentration of 1-90 μM in a 10 mM ammonium acetate buffer, whose pH was adjusted either by ammonium hydroxide or acetic acid. In order to avoid changes in the desired pH, caused by the adding of the sample buffer to the pH-adjusted buffer, highly concentrated protein solutions were used (454 μM for BLG and 323 μM for *Bos d 2*). Moreover, the possible pH changes were further tested on a larger scale by using the same dilution ratio of the sample buffer and the pH-adjusted buffer and by measuring the final pH of the combined solutions. To ensure a valid pH for each measurement, native mass spectra were first recorded from pH 7.0 to pH 3.0, and then from pH 7.0 to pH 9.0. Between the measurements, the sample transfer capillary was carefully flushed with the following pH buffer. Two or three parallel mass spectra were measured for each different pH value. The monomer-dimer ratio was obtained from the absolute intensities of the ions representing either the monomer (*P*) or the dimer (*P*_2_), by combining the intensities (*i*) of the ions appearing in different charge states. Assuming the same ionization and transmission efficiencies for the monomer and the dimer^23^, their intensity ratio sufficiently reflects the concentration ratio in solution. The same protocol was used when the dimerization of *Bos d 2* was studied as a function of the ionic strength. To determine *K*_D_ for the dimerization of both proteins, the protein concentration was varied from 0 to 90 μM, and the monomer-dimer ratio was obtained. The *K*_D_ value for protein dimerization can be defined as


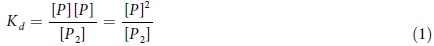


where [*P*] and [*P*_2_] are the equilibrium concentrations of the free protein monomer *P* and protein dimer *P*_2_, respectively. We can define the total protein (monomer) concentration [*P*]_total_ as


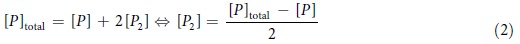


Inserting the above expression to the equation (1), we find that


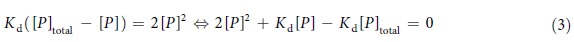


Finding roots for this second-order polynomial gives the expression


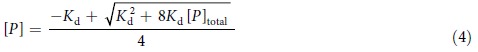


which defines [*P*] as a function of [*P*]_total_, if *K*_d_ is known. By fitting [*P*] as a function of [*P*]_total_, one can determine *K*_d_ in a wide range [*P*]_total_ by a nonlinear least-squares fitting procedure. A native mass spectrum for a transient dimer directly provides the intensity ratio of *P* and *P*_2_, which can be used to calculate [*P*] as


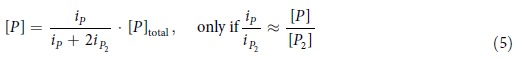


Total intensities for *P* and *P*_2_ (*i*_*P*_ and *i*_P2_) can be calculated as sums of peak intensities *i*_*z*_ over the charge state distribution (*z* = 1 to *n*) in the ESI-MS spectra. Fitting [*P*] against [*P*]_total_ using equation (4) gives *K*_D_. Equation (5) is valid only if ionization and ion transmission efficiencies are considered the same for *P* and *P*_2_. In the present and in our previous work^3^ we optimized all ion source and ion optics parameters to obtain similar transmission efficiency for both the monomer and the dimer. We used *Bos d 5* as a reference protein for which *K*_D_ is known. Liu *et al.* have previously shown that ionization efficiencies for the monomer and the dimer of *Bos d 5* are roughly the same^23^, hence the use of equation (5) is therefore warranted.

### Crystallisation of Bos d 2

The *rBos d 2* allergen was previously crystallised in the space group P2_1_2_1_2_1_ by using 10% (w/w) PEG6000, 0.1 M sodium acetate (pH 3.4–4.5) as a precipitant solution. The protein solution was concentrated up to 50 mg/ml for crystallisation experiments^7^. Alternative conditions for crystallisation of *rBos d 2* were searched for by using several commercially available crystallisation screens. Protein crystals belonging to space group P3_2_21 were obtained by using a 4.0 M sodium formate (pH 7.4) as a precipitant solution. Single crystals were obtained after 2 months of preparation of crystallisation drops. Protein was crystallised in the space group C2 by using the precipitant solution containing 2.5 M 1,6-hexanediol and 0.1 M sodium citrate (pH 6.4). The growing time for monoclinic crystal was 6 months. Both new crystal forms were obtained with a hanging-drop vapour-diffusion method at 293 K. Crystallisation drops were prepared by mixing 2 μl of protein solution (9 mg/ml in 50 mM ammonium acetate buffer, pH 6.7) and 2 μl of precipitant solution.

For the data collection, P3_2_21 crystals were cryoprotected with a 3.6 M sodium formate, 10% glycerol solution before freezing them in liquid nitrogen. The high hexanediol concentration of the precipitant solution enabled the direct transfer of C2 crystals from the crystallisation drops to liquid nitrogen. Both diffraction data sets were collected by using synchrotron radiation at the ESRF, Grenoble, France. The P3_2_21 crystal diffracted to a resolution of 1.75 Å on beamline ID29. For a complete data set, 900 diffraction images with a 0.1° oscillation range were collected with a Pilatus 6M pixel detector (Dectris Ltd). The diffraction data from the C2 crystal were collected on beamline ID14-4. The crystal diffracted to a resolution of 1.4 Å and the reflections were recorded with a Q315r ADSC detector. The crystal was rotated 180° with an oscillation angle of 0.5°. The data sets were processed with a XDS program and scaled and merged with XSCALE^32^.

### Structure determination and refinement

The packing of protein molecules in the new crystal forms was studied by using the structure of *rBos d 2* (PDB code 1BJ7) as a search model. The molecular replacement, carried out with the program Phaser^33^ as a part of the CCP4 suite^34^, indicated that both P3_2_21 and C2 crystal forms contained one *rBos d 2* molecule in an asymmetric unit. The solvent content of the crystals was 72% and 48%, respectively. The protein models were built manually with the program *Coot*^35^ and refined with the program PHENIX^36^. The protein model in the space group P3_2_21 was refined to final R_work_ and R_free_ values of 21.1% and 23.2%, respectively. The corresponding values for the *rBos d 2* model in the space group C2 were 19.6% and 21.4%. The final structures were validated with the program Procheck. The data collection and refinement statistics are presented in Table 2. Protein structures have been deposited in the RCSB Protein Data Bank with the accession codes of 4wfu and 4wfv.

### Analysis of crystal structures

The coordinates for the lipocalin allergens were from the Protein Data Bank (www.rcsb.org). PDBePISA (Proteins, Interfaces, Structures and Assemblies) (http://www.ebi.ac.uk/pdbe/pisa) was used to analyze protein-protein interfaces in crystals^37^. However, it should be noted that the program is not suitable for estimating the existence of transient dimers because it does not use protein concentration as a parameter. However, PISA is useful in analyzing protein-protein interfaces. In the estimation of the existence of a quaternary structure we used the area of monomer-monomer interface, the symmetry axis between monomers and the nature of interactions (hydrogen bonds, hydrophobic contacts) between monomers. The figures of the proteins were created with the PYMOL program [Delano, W. L. The PyMol Molecular Graphics System, www.pymol.org].

## Additional Information

**Accession codes:** The atomic coordinates and structure factors (codes 4WFV and 4WFU) have been deposited in the Protein Data Bank (http://wwpdb.org/).

**How to cite this article**: Niemi, M. H. *et al.* Dimerization of lipocalin allergens. *Sci. Rep.* **5**, 13841; doi: 10.1038/srep13841 (2015).

## Figures and Tables

**Table 1:** Determined crystal structures of lipocalin allergens.

**Animal**	**Source**	**Allergen**	**Amino acids**	**Resolution [Å]**	**PDB**	**Asym. unit**	**Space group**	**Oligomer**	**Interface1 [Å**^**2**^]	**Interface2 [Å**^**2**^]
Rat	urine	Rat n 1	162	2.90	2a2g	4	P2_1_2_1_2_1_	tetramer	708	457
Dog	saliva, dander	Can f 2	162	1.45	3l4r	1	C222_1_	tetramer	576	380
								dimer	447	
Horse	saliva, dander, urine	Equ c 1	172	2.30	1ew3	1	P4_3_2_1_2	dimer	1025	
Cockroach	whole body	Bla g 4	130	1.75	4n7c	1	P4_1_2_1_2	dimer	1018	
Cow	milk	Bos d 5	142	1.80	1beb	2	P1	dimer	528	
Mouse	dander, urine	Mus m 1	162	2.40	1mup	1	P4_3_2_1_2	dimer	389	
Cockroach	whole body	Per a 4	154	2.80	3ebw	2	P4_1_	dimer	403	
Dog	saliva, dander	Can f 4	158	2.60	4odd	3	P2_1_2_1_2_1_	dimer	640	
Pigeon tick	saliva	Arg r 1	144	1.40	2×45	3	C2	dimer	530	
Cow	dander	Bos d 2	156	1.75	4wfu	1	P3_2_21	dimer	389	

**Table 2:** Crystallographic data.

**Space group**	**C2**	**P3**_**2**_**21**
*Data Collection*		
Wavelength (Å)	1.0115	0.9763
Resolution range (Å)	50–1.4 (1.5–1.4)	50–1.75 (1.85–1.75)
Unit cell parameters		
a, b, c (Å)	125.9, 36.6, 36.5	75.5, 75.5, 95.2
α, β, γ(°)	90.0, 95.9, 90.0	90.0, 90.0, 120.0
No. of reflections	110938 (15382)	155026 (23674)
Unique reflections	31792 (5381)	31990 (4807)
Completeness (%)	96.9 (88.8)	99.4 (99.1)
Average I/σ	22.29 (4.17)	19.56 (2.64)
R_obs_ (%)	3.9 (29.9)	3.5 (66.7)
R_meas_ (%)	4.4 (36.8)	3.9 (74.7)
*Refinement and validation*		
No. of reflections, R_cryst_/R_free_	31789/1589	31890/1595
R_work_/R_free_ (%)	19.6/21.4	21.1/23.2
No. of protein atoms	1248	1248
No. of waters	67	90
Average B factors (Å^2^)		
Wilson	12.0	33.3
All atoms	14.1	38.1
Protein	13.7	37.6
Water	20.4	45.3
Rms deviations		
Bond lengths (Å)	0.006	0.006
Bond angles (°)	1.081	0.978
*Ramachandran plot*		
Most favoured (%)	89.6	88.0
Additional allowed (%)	10.4	12.0
Generously allowed (%)	0	0
Disallowed (%)	0	0
PDB code	4WFV	4WFU
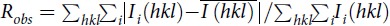		
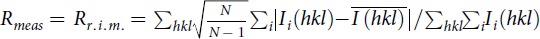		
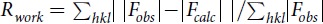		
